# Targeted proteomics and specific immunoassays reveal the presence of shared allergens between the zoonotic nematodes *Anisakis simplex* and *Pseudoterranova decipiens*

**DOI:** 10.1038/s41598-022-08113-3

**Published:** 2022-03-08

**Authors:** Ganna Saelens, Sören Planckaert, Victoria Martínez-Sernández, Florencio M. Ubeira, Bart Devreese, Sarah Gabriël

**Affiliations:** 1 Department of Translational Physiology, Infectiology and Public Health, Faculty of Veterinary Medicine, Ghent University, Salisburylaan 133, 9820 Merelbeke, Belgium; 2 Laboratory for Microbiology, Department of Biochemistry and Microbiology, Ghent University, K. L. Ledeganckstraat 35, 9000 Ghent, Belgium; 3 Department of Microbiology and Parasitology, Faculty of Pharmacy, Universidad de Santiago de Compostela, Praza do Seminario de Estudos Galegos, 15705 Santiago de Compostela, Spain; 4 Instituto de Investigación en Análisis Químicos y Biológicos (IAQBUS), Universidad de Santiago de Compostela, 15705 Santiago de Compostela, Spain

**Keywords:** Parasitology, Parasite biology, Food microbiology

## Abstract

The family Anisakidae, mainly represented by *Anisakis simplex* s.l. and *Pseudoterranova decipiens*, encompasses zoonotic nematodes infecting many marine fish. Both are responsible for gastrointestinal disease in humans after ingestion of a live larva by consumption of undercooked fish, and, in the case of *A. simplex*, an allergic reaction may occur after consuming or even handling infected fish. Due to its phylogenetic relatedness with *A. simplex*, few studies investigated the allergenic potential of *P. decipiens*, yet none of them focused on its excretory/secretory (E/S) proteins that easily get missed when working solely on extracts from crushed nematodes. Moreover, these E/S allergens remain behind even when the larva has been removed during fish quality processing. Therefore, the aim was to investigate if *Anisakis-*like allergens could also be detected in both crushed and E/S *P. decipiens* protein extract using targeted mass spectrometry analysis and immunological methods. The results confirmed that at least five *A. simplex* allergens have homologous proteins in *P. decipiens;* a result that emphasizes the importance of also including E/S protein extracts in proteomic studies. Not only *A. simplex*, but also *P. decipiens* should therefore be considered a potential source of allergens that could lead to hypersensitivity reactions in humans.

## Introduction

The presence of parasites in marine fish poses a serious economic and public health concern for the fishing industry worldwide. The family Anisakidae encompasses zoonotic nematodes with a global distribution and a complex lifecycle involving marine mammals, fish, molluscs and planktonic crustaceans. Humans may become accidental dead-end hosts after consumption of raw or undercooked fish or seafood products that contain a viable third-stage larva (L3), which may result into a mild to severe pathology. This foodborne disease, collectively named anisakidosis, causes gastrointestinal symptoms specifically characterized by acute and transient nausea, vomiting, diarrhea and epigastric pain within 1–12 h after consumption^1^. The main anisakid species causing the disease are *Anisakis simplex* sensu stricto (s.s.), *A*. *pegreffii*, *Pseudoterranova decipiens* and *Contracaecum osculatum*, which have also been the most studied^2^.

Additionally, human health may be compromised by an allergic reaction to allergens of *A. simplex* and *A. pegreffii* with clinical symptoms ranging from urticaria, angioedema, asthma, conjunctivitis, to even an anaphylactic shock. Also, due to the thermostable characteristics of several *Anisakis* allergens, some authors consider that an allergic reaction can also be elicited in sensitized persons by the accidental consumption of dead larvae in frozen/cooked fish or traces present in highly processed fishery products^3^, although this hypothesis was never convincingly demonstrated^4^. Finally, and different from other food allergies, *Anisakis* allergy can also be provoked by skin contact or inhalation^5^. As such, not only people consuming fish, but also all the globally 59.6 million people working in the fish industry can potentially be exposed to anisakid allergens^6^.

To date, numerous studies investigating the allergic properties of *A. simplex* and *A. pegreffii* have been performed. However, to our knowledge, the allergic potential of the commonly occurring *P. decipiens* that is phylogenetically closely related to the *Anisakis* genus has been neglected with only three studies directing immediate attention to it. In 2017, Ludovisi et al. showed that intraperitoneal infection of mice with live *P. decipiens* L3 could induce a mixed T helper type (Th) 1/Th2 response and make mice react (i.e., irritability and reduced activity) to a subsequent oral challenge with *P. decipiens* proteins^7^. An allergenic potential of *P. decipiens* was also supported by a proteomic study by Kochanowski, et al., who revealed the presence of at least eight *A. simplex*-like allergens in crushed *P. decipiens* extract and predicted another 28 putative allergens in both *A. simplex* and *P. decipiens* by use of high-resolution liquid chromatography coupled with tandem mass spectrometry (LC–MS/MS)^8^. Such LC–MS/MS method had also been previously applied by Carrera, et al. who identified one shared allergen (Ani s 9) between both nematode species^9^. While these studies already provide considerable evidence for an allergenic potential of *P. decipiens*, none of them focused on its excretory/secretory (E/S) proteins that may easily get missed when working solely on protein extracts from crushed nematodes. In fact, these E/S allergens should not be neglected as they remain behind even when the larva has been removed during the fish quality processing^10^.

Several detection methods for anisakid larvae and their allergens in fish have been developed with DNA-based methods and immunoassays remaining the most popular. However, despite their simplicity, the detection of anisakid DNA does not necessarily mean the presence of the proteins responsible for allergic reactions. Immunoassays on the other hand, often struggle with poor availability and quality of monoclonal/polyclonal antibodies. As a result, researchers have grasped for both discovery and targeted proteomics in which mass spectrometry is used to identify and detect a large number of anisakid proteins present in a sample in one single analysis. Especially targeted proteomics, such as multiple reaction monitoring (MRM) methods, have gained popularity as it only detects a preselected group of analytes of interest, rather than detecting all proteins in a mixture as is the case for discovery-based proteomics. This not only enhances sensitivity and reproducibility, but also the selectivity of the analysis by increasing the chance of detecting low-abundance proteins in highly complex mixtures^11^. Moreover, while detecting hidden allergens in food products is essential to protect the allergic population, the demand for a quantitative proteomic approach has been steadily rising as this assesses the risks associated with specific foods more accurately^12^. Here again, MRM ideally complements discovery proteomics by quantifying less-abundant proteins with high accuracy in a whole bulk of host cell proteins in the product^13^.

Considering these advantages that targeted proteomics offer, and the scarce knowledge on putative allergens in *P. decipiens,* in this study an LC–MS/MS method for *A. simplex* allergens by targeted MRM was developed and applied to both crushed and E/S protein extracts from *P. decipiens*. As such, possible shared allergens between both anisakid species could be detected simultaneously. Complementarily, and as proof of concept, the presence of Ani s 7-like molecules in *P. decipiens* was investigated using immunological methods.

## Materials and methods

### Preparation of *Anisakis simplex *sensu stricto and *Pseudoterranova decipiens* sensu stricto protein extracts

Live *Anisakis simplex* s.s. and *Pseudoterranova decipiens* s.s. third stage larvae (L3) were collected from the visceral cavity of European seabass (*Dicentrarchus labrax*) freshly caught from the North Sea. A total of 110 L3 *A. simplex* s.s. and 42 L3 *P. decipiens* s.s. were purified by six successive washing steps with sterile phosphate-buffered saline (PBS) pH 7.4 upon preparation of both E/S protein extracts (ESP) and crushed whole worm protein extracts (CrP). Species identification was performed by polymerase chain reaction-restriction fragment length polymorphism (PCR–RFLP) analysis as indicated in the section below. To obtain ESP, 100 and 40 washed *A. simplex* s.s. and *P. decipiens* s.s. L3 larvae, respectively, were cultured in separate sterile Petri dishes containing 20 mL of PBS and incubated at 37 °C in 5% CO_2_. Four hours post-incubation, the culture medium containing the E/S proteins was collected and concentrated by centrifugation (4032 *g*, 4 °C) for 170 min using Vivaspin 15R centrifugal concentrators (molecular weight (MW) cut-off 2000) (Vivaproducts, Littleton, USA). Both ESPs were stored at − 80 °C with addition of protease inhibitor (Sigma-Aldrich, Missouri, USA) until assayed. For the preparation of CrP, fresh and live *A. simplex* s.s. (*n* = 10) and *P. decipiens* s.s. (*n* = 2) larvae were crushed by use of a pestle in sterile potter tubes with 1 mL PBS (*A. simplex* s.s.) or 2 mL PBS (*P. decipiens* s.s.), both containing protease inhibitor. The mixture was subsequently centrifuged (15,000×*g*, 4 °C) for 20 min, and the supernatants were diluted in glycerol (1:1) before storage at − 20 °C until assayed. Two independent biological replicates of each parasite extract were prepared. Protein concentrations of all extracts were determined using the Pierce Coomassie Bradford Assay kit (Thermo Fisher, Massachusetts, USA).

### Anisakidae identification

Species identification occurred initially morphologically according to Petter and Maillard^14^, with subsequent PCR–RFLP analysis as described by the European Reference Laboratory for Parasitology^15^. Previous studies showed that this technique is able to identify larvae of *A. simplex* s.s., *A. pegreffii*, *A. simplex*/*A. pegreffii hybrid* genotype, *P. decipiens* s.s., and *Hysterothylacium* spp.^16^. For molecular identification of the larvae destined for CrP samples, a small larval piece was cut from each worm prior to crushing, whereas for the L3 destined for ESP, a larval piece was only taken after E/S protein production as the larvae had to remain intact for this. Subsequently, DNA was extracted from the larval piece, and the internal transcribed spacer region was amplified using the primers NC5 (5’-GTAGGTGAACCTGCGGAAGGATCAT-3’) and NC2 (5’-TTAGTTTCTTTTCCTCCGCT-3’). PCR samples were then digested with the endonucleases *Hin fI* and *Hha I*, and restriction fragments were separated by gel electrophoresis. After RFLP-PCR, the band patterns obtained from all samples classified as *A. simplex* s.s., and *P. decipiens* s.s., respectively.

### Tryptic digestion for LC–MS/MS analysis

In the sample preparation for the LC–MS/MS analysis, 10 µg of the protein sample extracts (*A. simplex* and *P. decipiens* ESP and CrP) were dried by SpeedVac centrifugation, solubilized in 50 mM NH_4_HCO_3_ spiked with bovine serum albumin (1:250 concentration ratio to protein extract), and denatured by heating to 80 °C for 10 min. The solution was subsequently reduced with 50 mM dithiothreitol (DTT) (10 min at 60 °C) and alkylated with iodoacetamide (20 min at room temperature (RT)) in the dark before digestion with trypsin overnight at 37 °C (1:50 w/w). Digested samples were then acidified with 8% HCOOH (30 min at 37 °C), until a first peptide purification step using Bond Elut OMIX C18 pipette tips (Crawford Scientific Ltd, Lanarkshire, UK) was performed. Here, the samples were first acidified to 1% HCOOH and the OMIX tips pre-washed with 0.1% HCOOH in acetonitrile (ACN) and ultrapure H_2_O (80:20, v/v). After pre-equilibration of the tips with 0.1% HCOOH in ultrapure H_2_O, peptides were captured on the C18 resin in the tips, washed with 0.1% HCOOH in ultrapure H_2_O, and eluted with 0.1% HCOOH in ACN and ultrapure H_2_O (60:40, v/v). Finally, the eluate was dried by SpeedVac centrifugation and stored at − 20 °C until LC–MS/MS analysis (see below).

### In-silico peptide selection in Skyline

For the detection of *A. simplex* allergens during liquid chromatography-mass spectrometric multiple reaction monitoring (MRM) analysis, unique peptides for each allergen were selected. This was performed in the freely available Skyline application (ver. 20.6, 64-bit, Seattle, USA, www.skyline.ms) in which precursor peptides and their fragment ions specific for each allergen were predicted by *in-silico* digestion^17^. The following parameters were set in “Peptide Settings”: *enzyme*: trypsin; *peptide length*: 8–25 amino acids long; *missed cleavages*: none; *modifications to be excluded*: peptides containing methionine, peptides containing lysine or arginine followed by proline; *structural modifications*: carbamidomethyl. For MRM-analysis the following parameters were set in “Transition settings”: *fragment ion charges*: 1; *ion type*; y. The sequences from 15 World Health Organization and International Union of Immunological Sciences (WHO/IUIS)-recognized *A. simplex* sensu lato (s.l.) allergens were obtained from Universal Protein Resource (UniProt) (http://uniprot.org), and proteotypicity of each predicted precursor peptide was verified using Unipept (www.unipept.ugent.be)^18^. Detection of allergens was only considered if their precursor peptides were proteotypic within *A. simplex* s.s. and when a minimum of two precursor peptides per allergen and three fragment ions per precursor peptide were available. Finally, retention times for each precursor peptide were experimentally obtained via test runs on *A. simplex* ESP and CrP, and imported in MassLynx software (ver. 4.2, Waters Corporation, Massachusetts, USA) for method creation (scheduled MRM).

### Targeted proteomic analysis using UPLC-Triple Quadrupole-MS/MS

Equal amounts of tryptic peptides (ESP and CrP) from both *A. simplex* s.s. and *P. decipiens* s.s. were analyzed by UPLC-MS/MS in MRM mode using a Waters NanoAcquity M-Class UPLC and an IonKey source connected to a Waters Xevo TQ-S triple quadrupole mass spectrometer (Waters Corporation). The IonKey source contained a 150 μm × 100 mm, 1.8 μm HSS T3, iKey separation device.

First, a phosphorylase B standard solution was added to each dried sample (10 fmol/µL sample, phosphorylase B diluted in H_2_O/ACN/HCOOH (97:3:0.1, v/v/v)) for UPLC-MS/MS performance evaluation. Each peptide solution was then transferred to an MS-vial from which five µL (0.5 µg) was injected and trapped (2 min, 15 µL/min, 3% ACN/0.1% HCOOH) onto a 300 μm × 50 mm, 5 μm, 100 Å Acquity UPLC M-Class Symmetry C18 Trap Colum (Waters) before separation on the iKey using a 3 to 50% ACN gradient for 10 min (2 µL/min flow rate). The outlet of the column was connected to the electrospray ionization source of the Waters Xevo TQ-S mass spectrometer with a capillary voltage of 3.6 kV, a cone voltage of 35 V and source temperature of 120 °C. The mass spectrometer was run in MRM mode with quadrupole (Q) 1 selecting precursor ions in a +/− 0.1 amu mass window, Q2 fragmenting them by collision induced dissociation (CID), and Q3 detecting the resulting fragment ions of interest. Data were acquired with the developed MRM mode, uploaded into Skyline for data analysis, and, subjected to a Savitsky-Golay smoothing. Finally, chromatograms with transition peaks for each precursor peptide from each allergen were manually verified. Default protein detection criteria included a minimum of two peptides per protein, minimum three fragment ions per peptide, and hence a total of six transitions per protein.

### ELISA determinations

Considering that the Ani s 7 allergen has internal sequence repeats which are recognized by monoclonal antibody (mAb) UA3^19^, this mAb was used as both capture (non-labeled) and detection antibody (labeled with FITC) in an enzyme-linked immunosorbent assay (ELISA) prototype kit (Inmunogal SL, Santiago de Compostela, Spain) to detect the presence of Ani s 7-like allergens in CrPs from *A. simplex* s.s. and *P. decipiens* s.s. and ESP from *P. decipiens* s.s. The detection limit of the assay was 40 ng/mL and 2.5 µg/mL, respectively, using recombinant truncated tAni s 7 and *Anisakis* CrP as standards (see Supplementary Fig. S1). The procedure was performed following the instructions provided with the kit. Briefly, 100 µL of each *A. simplex* s.s. CrP, *P. decipiens* s.s. CrP, and *P. decipiens* s.s. ESP were added at two protein concentrations (Table 3), and in duplicate to the wells of the ELISA plate coated with mAb UA3 and incubated for 30 min at RT while shaking (750 rpm). A CrP extract from *Ascaris suum* was used as negative control. After incubation and washing, the plate was aspirated and 100 µL fluorescein isothiocyanate (FITC)-labelled mAb UA3 alone or mixed with the inhibitor peptide (see below), was added to each well. After 20 min of incubation at RT while shaking (750 rpm) and further washing, bound FITC-mAb UA3 conjugate was detected by incubation for 20 min at RT under shaking (750 rpm) with horseradish peroxidase (HRP)-labelled rabbit anti-FITC polyclonal antibodies. Following a final wash, each well was incubated in the dark for 10 min with 100 µL TMB solution as a substrate for peroxidase. The color development was stopped by the addition of 100 µL 0.2 M H_2_SO_4_ and optical density (O.D.) was measured at 450 nm within 30 min after adding the stop solution. Samples were considered positive when their O.D. values exceeded 0.1 after subtraction of the O.D. value obtained for the negative control.

For competitive ELISA (cELISA), the synthetic peptide “CVQKYGTEFCNK” (P3, recognized by mAb UA3) was mixed at the molarities indicated in Table 3 with FITC-labelled mAb UA3 before adding it to the ELISA plate.

### SDS-PAGE and immunoblotting analysis

Both *A. simplex* and *P. decipiens* CrP extracts (10 µg per lane) were analyzed by sodium dodecyl sulfate polyacrylamide gel electrophoresis (SDS-PAGE) using precast gradient (8–16%) SurePAGE 10 × 8 gels (GenScript Biotech, Leiden, Netherlands). The proteins were separated under reducing conditions using MOPS as running buffer and 4 × LDS with DTT as sample buffer. PageRuler Plus prestained protein ladder (10–250 kDa; Thermo Fisher Scientific, Madrid, Spain) was used as MW marker. The electrophoresis was carried out at constant 200 V until front LDS markers reach the end of the gel.

Following electrophoresis, the proteins contained in the SDS-PAGE gel were blotted onto a 9.5 × 6.5 polyvinylidene fluoride (PVDF) membrane in an FSD-20 semi-dry blotter (Thermo Fisher Scientific) at 15 V for 60 min at RT. Twelve filter papers of the same size as the PVDF membrane (six for the cathode and six for the anode) were impregnated with the electrode buffer (48 mM Tris, 39 mM glycine, 0.037% SDS and 20% methanol; pH 9.2) and then used for proteins transfer. After blotting, the PVDF membrane was washed with distilled water and blocked with PBS buffer containing 0.05% Tween-20 (PBS-T) and 1.5% sodium caseinate for 2 h at RT. In parallel, the SDS-PAGE gel used for blotting was washed with distilled water and stained with BlueSafe (Nzytech, Lisboa, Portugal) to reveal the remaining proteins.

After blocking, the PVDF membrane was dried at RT, cut into strips, and stored at – 20 °C before subsequent use. To reveal the presence of Ani s 7 (*A. simplex* CrP) and Ani s 7-like (*P. decipiens* CrP) antigens, a set of each blotted PVDF strips was incubated with mAb UA3 diluted 1:2000 in PBS-T containing 1% non-fat dry milk (PBS-T-DM) alone or in PBS-T-DM containing 50 μM of the synthetic peptide P3. The incubation procedure was performed under slow shaking for 1 h at RT. Then, the strips were washed with PBS-T and incubated with goat anti-mouse IgG HRP conjugate (Bio-Rad, Madrid, Spain; dilution 1:1600) under the same conditions as above. Afterwards, the strips were washed with PBS-T and the immune reaction was revealed by incubation with the enzyme substrate (SigmaFAST 3,3′-Diaminobenzidine tablets) prepared according to manufacturer instructions (Merck, Madrid, Spain).

## Results

### Selection of precursor peptides and fragment ions specific for each allergen

To detect *A. simplex* allergens in the prepared ESP and CrP, specific proteotypic peptides and their fragment ions were first selected by *in-silico* digestion using the Skyline software. For this, the amino acid sequences from 15 WHO/IUIS-reviewed *A. simplex* (s.l.) allergens (Ani s 1.0101, Ani s 2.0101, Ani s 3.0101, Ani s 4.0101, Ani s 5.0101, Ani s 6.0101, Ani s 7.0101, Ani s 8.0101, Ani s 9.0101, Ani s 10.0101, Ani s 11.0101, Ani s 11.0201, Ani s 12.0101, Ani s 13.0101, and Ani s 14.0101) were obtained from UniProt and imported into the Skyline software for selection of precursor peptides according to the criteria described before.

Apart from two allergens (Ani s 10.0101 and Ani s 6.0101) for which only one precursor peptide could be predicted, all allergens had at least two predicted precursor peptides each containing at least three fragment ions. When tested for proteotypicity, nearly all predicted precursor peptides were proteotypic for the genus *Anisakis* as validated by homology searching with Unipept. However, several predicted precursor peptides of Ani s 2.0101 and Ani s 3.0101, revealed 100% amino acid sequence identity with peptides from muscle proteins from crustaceans, insects and other nematode species (i.e., *Ascaris* spp., *Ancylostoma* spp., *Angiostrongylus* spp., *Onchocerca* spp., *Strongyloides* spp., *Trichinella* spp., *Toxocara* spp., *Trichuris* spp., *Wuchereria* spp.). Like for allergens Ani s 1.0101, 2.0101, 9.0101, 12.0101, and 13.0101, though proteotypic to the *Anisakis* genus, homology searching in Unipept for several predicted precursor peptides revealed 100% amino acid sequence identity to a peptide in the phylogenetically closely related *Anisakis pegreffii*. However, since the anisakids used for the protein preparation had been subjected to molecular species confirmation by PCR–RFLP, it was decided to include the peptides to the MRM method, yet they should not be used for specific detection of *A. simplex* s.s. allergens during food quality control. Finally, allergens Ani s 11.0101 and Ani s 11.0201, though proteotypic to the *Anisakis* genus, shared two from the three predicted precursor peptides which hence were not proteotypic within the *A. simplex* s.s. species for its allergen and were excluded. As a result, Ani s 11.0101 and Ani s 11.0201, together with Ani s 6.0101 and Ani s 10.0101, were left with only one precursor peptide and hence would not fulfill the default protein detection criteria (i.e., minimum of two precursor peptides per protein and a minimum of three fragment ions per precursor peptide). Consequently, the remaining 11 allergens (Ani s 1.0101, Ani s 2.0101, Ani s 3.0101, Ani s 4.0101, Ani s 5.0101, Ani s 7.0101, Ani s 8.0101, Ani s 9.0101, Ani s 12.0101, Ani s 13.0101, and Ani s 14.0101) were included for allergen detection with Triple Q MS/MS analysis. Each allergen with its UniProt code, biological information (function, amino-acid length, molecular weight, etc.), and number of precursor peptides with minimum three fragment ions, is listed in Table 1. Additionally, a list of the proteins with all predicted transitions (precursor peptides and fragment ions), m/z ratios, and collision energies for the method development and analysis by Triple Q MS/MS has been provided in Supplementary Table S1.

**Table 1:** Overview of *Anisakis simplex* allergens with its UniProt code, biological information, and number of precursor peptides fulfilling the Skyline selection criteria, being proteotypic within the *A. simplex* species, and containing a minimum of three fragment ions.

Allergen name with UniProt code	Allergen information (major/minor, biological function, Excretory/Secretory (E/S), somatic or cuticular)	Amino-acid length	Molecular weight (kDa)	Number of precursor peptides proteotypic within *Anisakis simplex* (s.s.)	Fulfilled the default protein inclusion criteria (Yes/No)
Ani s 1.0101 (Q7Z1K3)	Major, Serine protease inhibitor, E/S	194	24	5	Yes
Ani s 2.0101 (Q9NJA9)	Major, Paramyosin, E/S	869	97	13	Yes
Ani s 3.0101 (Q9NAS5)	Minor, Tropomyosin, Somatic	284	41	9	Yes
Ani s 4.0101 (Q14QT4)	Minor, Cystatin, E/S	115	9	3	Yes
Ani s 5.0101 (A1IKL2)	Minor, SXP/RAL-2 family protein, E/S	152	15	5	Yes
Ani s 6.0101 (A1IKL3)	Minor, Serine protease inhibitor, E/S	84	10	1	No
Ani s 7.0101 (A9XBJ8)	Major, Glycoprotein, E/S	1096	139	10	Yes
Ani s 8.0101 (A7M6Q6)	Minor, SXP/RAL-2 family protein, E/S	150	15	7	Yes
Ani s 9.0101 (B2XCP1)	Minor, SXP/RAL-2 family protein, E/S	147	14	6	Yes
Ani s 10.0101 (D2K835)	Minor, Unknown function, somatic or cuticular	231	21	1	No
Ani s 11.0101 (E9RFF3)	Major, Unknown function	307	27	1	No
Ani s 11.0201 (E9RFF5)	Major, Unknown function	287	28	1	No
Ani s 12.0101 (E9RFF6)	Major, Unknown function	295	31	5	Yes
Ani s 13.0101 (A0A221C790)	Major, Hemoglobin, Somatic	309	37	11	Yes
Ani s 14.0101 (A0A0S3Q267)	Major, N-terminal, partial sequence, Unknown function	217	24	7	Yes

### Detection of several allergens in both protein extracts using the UPLC-MS/MS in MRM mode

**Figure 1:**
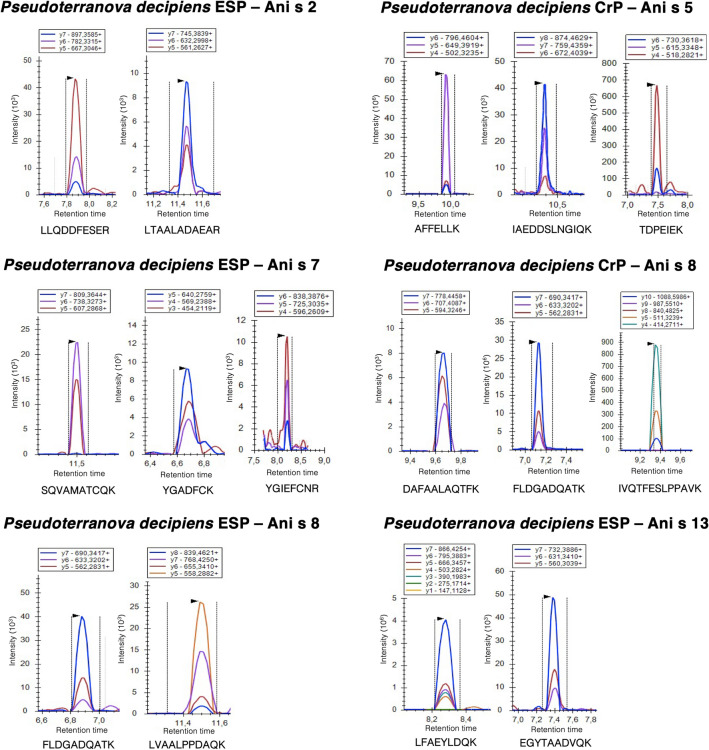
Transition peaks on Skyline for precursor peptides from Ani s 2.0101, Ani s 5.0101, Ani s 7.0101, Ani s 8.0101, and Ani s 13.0101 detected in *Pseudoterranova decipiens* excretory/secretory and crude worm protein extracts (ESP and CrP, respectively). Each color represents a different transition (i.e., precursor peptide/fragment ion pair).

Using the UPLC-MS/MS in MRM mode, precursor peptides from in total five *A. simplex*-like allergens (Ani s 2.0101, Ani s 5.0101, Ani s 7.0101, Ani s 8.0101, and Ani s 13.0101) were detected in the ESP and/or CrP of *P. decipiens* s.s. (Table 2, Fig. 1)*.* Specifically, three precursor peptides from Ani s 5.0101 (IAEDDSLNGIQK, TDPEIEK and AFFELLK) were detected in *P. decipiens* s.s. CrP, while a total of seven precursor peptides from Ani s 13.0101 (EGYTAADVQK and LFAEYLDQK), Ani s 2.0101 (LTAALADAEAR and LLQDDFESER) and Ani s 7.0101 (YGIEFCNR, YGADFCK and SQVAMATCQK) were detected in *P. decipiens* s.s. ESP. In addition, two (FLDGADQATK and LVAALPPDAQK) and three (FLDGADQATK, DAFAALAQTFK and IVQTFESLPPAVK) precursor peptides respectively from Ani s 8.0101 were detected in both *P. decipiens* s.s. ESP and CrP. Also, one precursor peptide from Ani s 2.0101, Ani s 3.0101, Ani s 5.0101, Ani s 10.0101 and Ani s 13.0101 was detected in *P. decipiens* s.s. ESP and/or CrP extract, which does not meet the requirements for unambiguous protein identification. Finally, no precursor peptides for Ani s 4.0101, Ani s 6.0101, Ani s 9.0101, Ani s 11.0101, Ani s 11.0201, Ani s 12.0101, and Ani s 14.0101 were detected in any of the *P. decipiens* s.s. protein extracts.

**Table 2:** Overview of precursor peptides with their number of fragment ions from *Anisakis simplex* allergens detected in *A. simplex* and *Pseudoterranova decipiens* protein extracts (excretory-secretory and crude worm protein extracts).

Allergen name	Precursor peptides from *A. simplex* allergens with number of fragment ions detected in *A. simplex* s.s. ESP	Precursor peptides from *A. simplex* allergens with number of fragment ions detected in *A. simplex* s.s. CrP	Precursor peptides from *A. simplex* allergens with number of fragment ions detected in *P. decipiens* s.s. ESP	Precursor peptides from *A. simplex* allergens with number of fragment ions detected in *P. decipiens* s.s. CrP
Ani s 1.0101	TECQLPLDK (3) SGICLSFK (3)	SCDDQFCPEDAK (4) SGICLSFK (3)	None detected	None detected
Ani s 2.0101	DLEVALDEETR (3) LLQDDFESER (3)	DLEVALDEETR (3) ADLSVQLIALTDR (3) ADQAESSLNLIR (3) ISDLTSINSNLTAIK (3) LLQDDFESER (3) QAEADLEEAHVR (3)	LTAALADAEAR (3) LLQDDFESER (3)	DLEVALDEETR (3) ^a^
Ani s 3.0101	VQEAEAEVAALNR (3) SLEVSEEK (3)	IVELEEELR (3) ANTVESQLK (3) SLEVSEEK (3) VQEAEAEVAALNR (3)	None detected	VQEAEAEVAALNR (3) ^a^
Ani s 4.0101	None detected	None detected	None detected	None detected
Ani s 5.0101	IAEDDSLNGIQK (3) ^a^	IAEDDSLNGIQK (3) AFFELLK (3)	IAEDDSLNGIQK (3) ^a^	IAEDDSLNGIQK (3) AFFELLK (3) TDPEIEK (3)
Ani s 6.0101	None detected	None detected	None detected	None detected
Ani s 7.0101	SQVAMATCQK (3) ^a^	None detected	SQVAMATCQK (3) YGADFCK (3) YGIEFCNR (3)	None detected
Ani s 8.0101	FLDGADQATK (3) DAFAALAQTFK (3) ADAELTAIADDASLTLAAK (3)	FLDGADQATK (3) DAFAALAQTFK (3) ADAELTAIADDASLTLAAK (3)	FLDGADQATK (3) LVAALPPDAQK (4)	FLDGADQATK (3) DAFAALAQTFK (3)
Ani s 9.0101	GGAVQAEFNK (3) ^a^	GGAVQAEFNK (3) QLAAAFQALDPAVK (4)	None detected	None detected
Ani s 10.0101	ANEQAAEQQNIGVGGPGPVK (5) ^a^	ANEQAAEQQNIGVGGPGPVK (5) ^a^	None detected	ANEQAAEQQNIGVGGPGPVK (5) ^a^
Ani s 11.0101	None detected	None detected	None detected	None detected
Ani s 11.0201	None detected	GPLPIGGPGPVVSGSGIGR (4) ^a^	None detected	None detected
Ani s 12.0101	YGENCAELIK (3) ^a^	QCVTITGAPPVTIGGSGQYR (3) EQCIESQIVIR (3)	None detected	None detected
Ani s 13.0101	LFAEYLDQK (6) EGYTAADVQK (3) QAWLEIGK (3) EFSSEITK (3) SHSHLTEDEK (3)	LFAEYLDQK (6) EGYTAADVQK (3) QAWLEIGK (3) EFSSEITK (3)	LFAEYLDQK (3) EGYTAADVQK (3)	LFAEYLDQK (5) ^a^
Ani s 14.0101	None detected	YSESFCNR (3) TNPLNAQCR (3)	None detected	None detected

An allergen was considered present when a minimum of two proteotypic precursor peptides with each at least three fragment ions were detected.

*ESP* excretory-secretory protein extract, *CrP* crude worm protein extract.

^a^Presence of allergen uncertain; insufficient precursor peptides.

Regarding *A. simplex* s.l. databases, peptide precursors from at least nine reported allergens were detected in the *A. simplex* s.s. ESP and/or CrP extracts. Specifically, two or more proteotypic precursor peptides matching Ani s 1.0101 (SCDDQFCPEDAK and SGICLSFK), Ani s 2.0101 (ADLSVQLIALTDR, DLEVALDEETR, ADQAESSLNLIR, ISDLTSINSNLTAIK, LLQDDFESER and QAEADLEEAHVR), Ani s 3.0101 (IVELEEELR, SLEVSEEK, VQEAEAEVAALNR, and ANTVESQLK), Ani s 5.0101 (AFFELLK and IAEDDSLNGIQK), Ani s 8.0101 (ADAELTAIADDASLTLAAK, DAFAALAQTFK, FLDGADQATK and IVQTFESLPPAVK), Ani s 9.0101 (GGAVQAEFNK and QLAAAFQALDPAVK), Ani s 12.0101 (EQCIESQIVIR and QCVTITGAPPVTIGGSGQYR), Ani s 13.0101 (SHSHLTEDEK, QAWLEIGK, LFAEYLDQK, EGYTAADVQK and EFSSEITK), and Ani s 14.0101 (TNPLNAQCR and YSESFCNR) were found in *A. simplex* s.s. CrP. Similarly, two or more proteotypic peptides from Ani s 1.0101 (TECQLPLDK and SGICLSFK), Ani s 2.0101 (DLEVALDEETR and LLQDDFESER), Ani s 3.0101 (SLEVSEEK, VQEAEAEVAALNR), Ani s 8.0101 (ADAELTAIADDASLTLAAK, DAFAALAQTFK and FLDGADQATK), and Ani s 13.0101 (QAWLEIGK, LFAEYLDQK, EGYTAADVQK and EFSSEITK) were detected in *A. simplex* s.s. ESP. However, only one precursor peptide from allergens Ani s 5.0101, Ani s 7.0101, Ani s 9.0101, Ani s 10.0101, Ani s 11.0201, and Ani s 12.0101, was found in *A. simplex* s.s. ESP and/or CrP which does not meet the requirements for unambiguous protein identification. Finally, no precursor peptides from allergens Ani s 4.0101 and Ani s 11.0101 were detected in any of the *A. simplex* s.s. extracts. An overview of precursor peptides with their number of fragment ions from *A. simplex* allergens detected in *A. simplex* s.s. and *P. decipiens* s.s. protein extracts is presented in Table 2.

### Detection of Ani s 7-like molecules in *Pseudoterranova decipiens*

**Figure 2:**
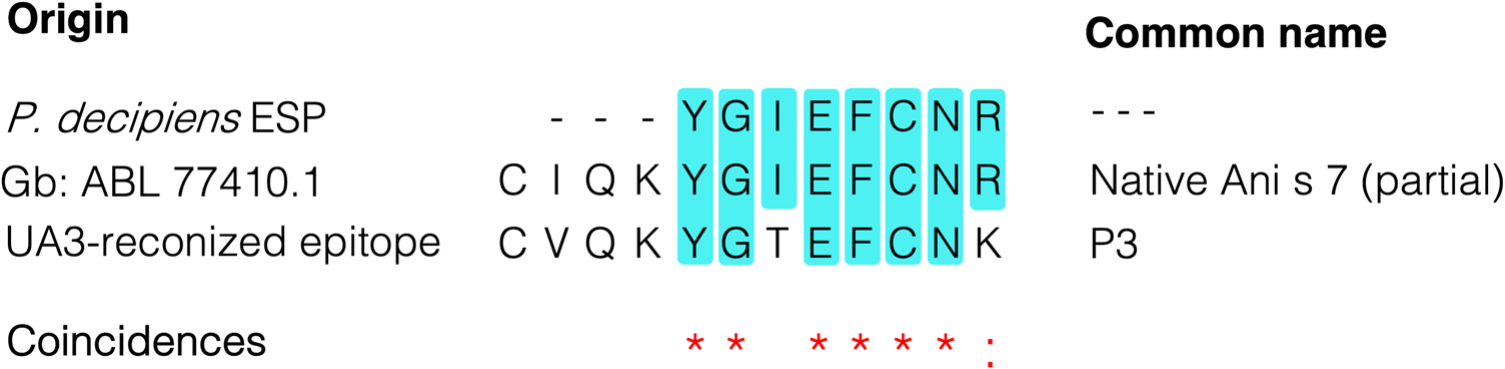
Alignment of the Ani s 7.0101 precursor peptide YGIEFCNR detected in the *Pseudoterranova decipiens* s.s. excretory-secretory protein extract (ESP) with the amino acid sequence from the protein ABL77410.1 (partial Ani s 7.0101) and the amino acid sequence (P3), both reported to be recognized by mAb UA3^19^.

Among the putative allergens detected by the UPLC-MS/MS analysis in *P. decipiens* s.s. preparations (Table 2), several precursor peptides matching partially the Ani s 7 allergen were found. Interestingly, one of such peptides corresponded to the “YGIEFCNR” sequence which seemed to have partial correspondence with amino acid sequences previously reported to be recognized by mAb UA3 on the Ani s 7 allergen^19^. As can be seen in the alignment shown in Fig. 2, this tryptic peptide shows 75% sequency identity (6/8) and 87.5% (7/8) similarity with two UA3-recognized sequences (i.e., P3 and the partial sequence of native Ani s 7.0101, respectively). Moreover, as Ani s 7 is the reported *Anisakis* specific allergen more frequently recognized by infected patients^20–22^, we investigated whether the mAb UA3 is able to recognize an Ani s 7-like molecule in *P. decipiens* s.s. preparations using a sandwich ELISA.

As can be seen in Table 3, all the investigated samples (*P. decipiens* s.s. CrP, *P. decipiens* s.s. ESP, and *A. simplex* s.s. CrP) tested positive in ELISA. Indeed, the O.D. values obtained for each extract exceeded largely the arbitrary cut-off value (O.D. = 0.1) established for the assay at all protein concentrations. Moreover, the binding of the mAb UA3 to all parasite extracts could be inhibited in the range of 59–97% by the UA3-recognized peptide P3 which demonstrated the specificity of the obtained O.D. signals. In contrast, the *A. suum* extract used as negative control tested negative, as expected.

**Table 3:** Overview of the prepared *Anisakis simplex* and *Pseudoterranova decipiens* protein extracts combined with their protein concentration, optical density (with and without inhibition with the *A. simplex* peptide P3), and test result.

Sample	Protein concentration (µg/mL)	P3 Molarity (µM)	O.D. (no inhibition)	O.D. (with inhibition)	% Inhibition	Test result
*A. simplex* CrP	500	100	1.37	0.42	69.04	Positive
	125	25	0.83	0.26	68.75	Positive
*P. decipiens* ESP	220	400	0.58	0.04	93.1 97.05	Positive
	125	200	0.34	0.01		Positive
*P. decipiens* CrP	500	100	0.68	0.19	86.19	Positive
*Ascaris suum* CrP	125 2200	25	0.25	0.10	59.22	Positive
		400	0.00	NA	NA	Negative

*NA* not applicable, *O.D.* optical density, *ESP* excretory/secretory protein extract, *CrP* crushed whole worm protein extract.

### Gel electrophoresis and Western blot analysis of *Anisakis simplex* and *Pseudoterranova decipiens* crude protein extracts

**Figure 3:**
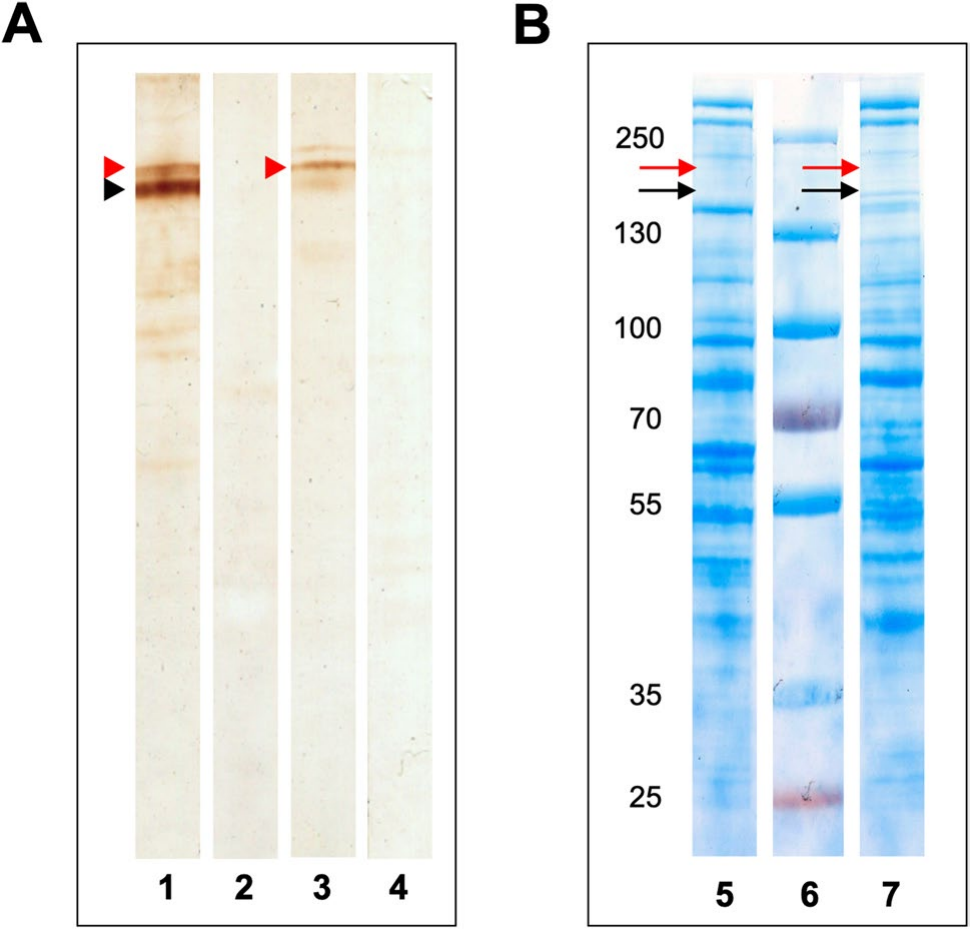
(**A**) Western blot analysis of *A. simplex* (lane 1) and *P. decipiens* (lane 3) crude protein extract with monoclonal antibody (mAb) UA3, and after inhibition with peptide P3 (lane 2 and 4, respectively). Arrowheads indicate the position of the common major band recognized by the mAb UA3 in both extracts. (**B**) BlueSafe-stained SDS-PAGE of *A. simplex* (lane 5) and *P. decipiens* (lane 7) crude protein extracts remaining proteins after blotting. Lane 6 shows the migration of the molecular weight marker corresponding to the values indicated at the left of the figure. Lanes in the figure were cropped from whole gel/blots images as shown in Figure S2 for a better definition of the high molecular weight region where mAb UA3 reacts with *Anisakis* antigens^23^.

To definitely confirm the presence of Ani s 7-like molecules in *P. decipiens* s.s. extracts, and to have an estimation of their MWs, a WB and WB-inhibition comparative analysis of *A. simplex* s.s. and *P. decipiens* s.s. CrP extracts was carried out. The results of the WB with mAb UA3 showed coherent results with the ELISA and confirmed the presence of at least one Ani s 7-like molecule in the *P. decipiens* s.s. CrP. Specifically, in both *A. simplex* s.s. and *P. decipiens* s.s. CrP, it was possible to observe a common major band recognized by the mAb UA3 (Fig. 3A, red arrowheads), although the major band recognized by mAb UA3 was less evident for the *P. decipiens* s.s. CrP extract (Fig. 3A, black arrowhead). As for the ELISA, inhibition of WB signal with peptide P3 revealed no bands for both extracts, which demonstrated the specificity of the assay. In Fig. 3B, the protein profiles of *A. simplex* s.s. and *P. decipiens* s.s. CrP which remained in the gel after blotting are demonstrated. It can be observed that both profiles are similar, but also that UA3-recognized antigens are minor as they could not be evidenced after gel staining (see red and black arrows). Lanes in Fig. 3 were cropped from whole gel/blot images as shown in Supplementary Fig. S2 for a better definition of the high MW region where mAb UA3 reacts with *Anisakis* antigens^23^.

## Discussion

*Anisakis simplex* s.s. and *A. pegreffii* are the only anisakid species currently associated with allergic responses caused by *Anisakis* infections or by inhalation of their allergens^24,25^. Despite a phylogenetically relatedness of *P. decipiens* with the *Anisakis* genus, only few studies examined its allergenicity and explored its proteome. Kochanowski, et al. already predicted 28 putative allergens and revealed the presence of Ani s 2.0101, 3.0101, 5.0101, 8.0101, and 9.0101 in *P. decipiens* CrP^8^, whereas Carrera, et al. similarly reported Ani s 9.0101 as a shared allergen between both anisakid species^9^.

Using LC–MS/MS spectrometry in targeted MRM mode, in this study we identified at least two precursor peptides with three or more fragment ions for 11 *Anisakis*-like allergens. An allergen was considered present when two predicted precursor peptides with each minimum three fragment ions, were detected. By solely subjecting *P. decipiens* CrP to the LC–MS/MS, only Ani s 5.0101 and 8.0101 allergen homologues were identified. This is a lower number compared to the study from Kochanowski, et al. in which also Ani s 2.0101, Ani s 3.0101, and Ani s 9.0101 homologues were detected in the *P. decipiens* CrP in a non-targeted proteomics experiment^8^. It should be noted that these authors used less stringent criteria to accept an identification. In addition, it has been suggested that the production of E/S, cuticular and somatic compounds in the third stage larvae varies depending on the temperature and the host from which it has been isolated^26,27^. As such, every prepared ESP and CrP is only a representation of the moment which may differ from one another. Nevertheless, when also analyzing the *P. decipiens* ESP in the present study, homologues of another four allergens (i.e., Ani s 2.0101, Ani s 7.0101, Ani s 8.0101, and Ani s 13.0101) were found from which two (i.e., Ani s 7.0101 and Ani s 13.0101) had not been previously detected in *P. decipiens*.

The inclusion of E/S proteins in proteomic/allergenic studies is important as the majority of the *Anisakis* E/S proteins are major allergens and hence recognized by more than 50% of the sensitized patients. In fact, the responses to nematode E/S antigens occur earlier and tend to be stronger than those induced by somatic antigens. This higher allergenic potency could be a result of their higher affinity to specific IgE compared to somatic allergens, and their potential to alter and/or suppress the human immune response system^28,29^. Also, as E/S proteins are released by the larvae during their migration through the body of fish, they remain behind after the larva is removed from the fish during quality inspection. Moreover, of all pepsin- and thermostable *A. simplex* allergens, most of them are present in E/S products and are not destroyed by cooking or freezing the fish^30^. Therefore, in the present study, both ESP and CrP from *A. simplex* and *P. decipiens* were analyzed by LC–MS/MS for comparative analysis.

Among the detected *P. decipiens* s.s. allergen homologues, Ani s 5.0101 and Ani s 8.0101 are both minor E/S allergens characterized as SXP/RAL-2 family proteins with up till to date unknown physiological functions. The predicted and detected precursor peptides DAFAALAQTFK and IVQTFESLPPAVK for Ani s 8.0101 from the present study had already been selected and successfully implemented by Faeste, et al. as suitable marker peptides for the presence of *A. simplex* in food and feed^31^. Ani s 7.0101 is also an E/S allergen of *A. simplex* but, unlike Ani s 5.0101 and Ani s 8.0101, it is categorized as one of the most important major allergens since nearly all infected patients show an allergic response against this allergen^19–22^. Apart from one precursor peptide of Ani s 7.0101 detected in the *A. simplex* s.s. ESP by the LC–MS/MS technology, two other Ani s 7.0101 precursor peptides were additionally detected in the *P. decipiens* s.s. in the ESP, but not the CrP. This could simply be explained by the E/S nature of Ani s 7.0101 which is expected to present in much lower concentrations in the CrP extract (see titration curve with CrP extract in Fig. S1) compared to the ESP. Additionally, as abovementioned, the larger body surface of *P. decipiens* may have resulted in a higher concentration of the allergen in the ESP extract compared to *A. simplex*.

Additionally, homologues of another two major allergens (Ani s 2.0101 and Ani s 13.0101) were discovered in the *P. decipiens* s.s. extract, both of which belonging to the somatic proteins^10^. Ani s 2.0101, also called paramyosin, is considered a panallergen due to its high similarities with muscle proteins from other nematodes, crustaceans, or insects and together with Ani s 1.0101 it is recognized by at least 88% of the sensitized people^32^. The second detected somatic allergen (Ani s 13.0101) is the *Anisakis* hemoglobin, which also presents high rates of recognition (81%) by sensitized patients and has the function to protect the parasite from immune responses of the host^33,34^. Similar as with Ani s 8.0101, Faeste, et al. selected marker peptides (LFAEYLDQK and HSWTTIGEEFGHEADK) for this allergen that were also predicted during our *in*-*silico* modelling and detected in the *P. decipiens* s.s. protein extract^31^. Furthermore, for another five allergens (Ani s 2.0101, Ani s 3.0101, Ani s 5.0101, Ani s 10.0101, and Ani s 13.0101), only one predicted precursor peptide was detected in the *P. decipiens* s.s. ESP and/or CrP which was not sufficient to fulfill protein detection criteria yet does not exclude their possible presence. In general, a protein with matches to only a single peptide sequence is exposed to a higher false positive discovery rate, however, this limitation has to be accepted when only one proteotypic peptide could be predicted (as was the case for the detected Ani s 10.0101)^35^. Lastly, but not least, it should be noted that in terms of allergenicity and protein sequence/structure, the finding of precursor peptides by mass spectrometry in our *P. decipiens* s.s. extracts, is only indicative for the presence of proteins which are homologous of the corresponding *Anisakis* allergens, i.e., *Anisakis*-like proteins. In other words, the fact that two proteins from different origin share one or more small fragment sequences, does not implicate that these proteins have the same primary structure, nor the same allergenic properties. However, in terms of food allergy safety, it is advisable to opt for a zero-risk approach and assume the presence of an allergen in the case of already one peptide match.

A potential limitation of the present LC–MS/MS analysis is that quantification was not performed, though this can be overcome in the future by adding isotope-labelled peptides as internal standards to the sample extracts. On the other hand, reliable and reproducible quantification of allergens in more complex matrices is often complicated by sensitivity issues due to possible matrix interference and the selected protein extraction and purification system. The latter in combination with the lack of scientifically substantiated threshold levels in allergenic individuals, has made the industry grasp for precautionary allergen labelling (e.g., statements such as ‘may contain traces of’) rather than implementing a validated quantification system.

An alternative to mass spectrometry methods, is the use of specific immunoassays. When immunoassays are provided with high quality antibodies (e.g., using monoclonal antibodies), analytes can be detected with a similar, or even better, sensitivity than targeted proteomics^11^. However, immunoassays remain frequently restricted to a single well-known protein, whereas MRM methods on Triple Q mass spectrometers may detect and quantify hundreds of proteins in a multiplexed fashion^12^. This explains why the latter methods are currently the common strategies applied for the detection and quantification of residues (allergens, proteins, parasites, etc.) in food and already had application in quantifying *A. simplex* allergens in fish and fish fillets^36–38^. Fortunately, immunological methods and mass spectrometry can be combined to target specific proteins in complex matrices, as has been proposed by several scientists^11,39^. So, taken the advantage of having an mAb (UA3) that recognizes some linear epitope sequences which include the sequence “YGIEFCNR” from Ani s 7.0101 (Fig. 2), two immunoassays were carried out complementarily to the LC–MS/MS analysis. Remarkably, the results from the ELISA/ELISA-inhibition and WB/WB-inhibition confirmed the presence of Ani s 7-like molecules in CrP extracts and E/S preparations from *P. decipiens*. Moreover, the fact that the antigen could be recognized in the cELISA using the same mAb UA3 also indicated that the Ani s 7-like molecules from *P. decipiens* have two or more UA3-recognized epitopes as occurs with *A. simplex*. However, this does not necessarily implicate that such epitope is immunodominant to humans infected with *P. decipiens* which can only be demonstrated by monospecific testing of human sera for *P. decipiens*. Additionally, as deduced from the different profiles of the recognized bands shown in the WB analysis (Fig. 3), it can also be assumed that the Ani s 7-like molecules from *P. decipiens* have structural differences with respect to the Ani s 7.0101 allergen. To determine the latter, and, whether or not *P. decipiens* Ani s 7-like molecules are antigenically cross-reactive with Ani s 7.0101, gene sequencing studies need to be carried out.

In summary, in the present study we confirmed that at least five WHO/IUIS-approved *A. simplex* allergens have homologous proteins in *P. decipiens*. While the allergenic potential of *P. decipiens* was already reported previously, our findings have contributed to this knowledge with two extra *A. simplex* homologous proteins being detected for the first time in *P. decipiens* protein extracts. As such, this nematode, which is highly present in several often-consumed marine fish species, should not only be considered a source for gastric infections (pseudoterranovosis), but also as a potential inducer of hypersensitivity reactions in humans. Nevertheless, further *in*-*vivo* and *in*-*vitro* investigations to confirm the allergenic potency of these molecules in *P. decipiens* are required.

## Supplementary Information

Supplementary Information.

## Data Availability

Apart from the MS/MS data, all data generated or analyzed during this study is included in this published article (and its Supplementary Information files). A summary of the MS/MS data is provided in Fig. 1 and Tables 1 and 2, and all raw MS/MS data (including the transition list) are available in the PeptideAtlas repository (http://www.peptideatlas.org/repository/) using the dataset identifier PASS01718 (made available 01/01/22).
