# Supplementary material for: Targeted proteomics and specific immunoassays reveal the presence of shared allergens between the zoonotic nematodes Anisakis simplex and Pseudoterranova decipiens
Source: Sci Rep. 2022 Mar 8;12:4127. doi: 10.1038/s41598-022-08113-3 (PMC8904469; doi:10.1038/s41598-022-08113-3)

Table 1S. *Anisakis simplex* allergens with their predicted transitions, m/z ratios, and collision energies for multiple reaction monitoring validation.

[a] Precursor peptide not proteotypic to the *Anisakis* genus.
[b] Precursor peptide proteotypic to the *Anisakis* genus, but showing 100% amino acid sequence identity to a peptide in *Anisakis pegreffii*.
[c] Precursor peptide proteotypic to the Anisakis genus, but not within the *A. simplex* species.

Allergen name with UniProt code	Precursor peptide sequence	Precursor (m/z)	Fragment ion (m/z)	Collision energy (V)
Ani s 1.0101 (Q7Z1K3)	TECQLPLDK	552.273706	873.449865	19
			713.419216	
			585.360639	
			472.276575	
	SGICLSFK	456.236395	767.412023	16
			654.327959	
			494.29731	
	YTGCGGNANR [a]	535.227622	805.33696	18
			748.315496	
			588.284848	
	CPNGYQCK	513.710219	866.382513	19
			769.32975	
			655.286822	
			598.265358	
	SCDDQFCPEDAK	736.276848	1109.456801	26
			994.429858	
			866.37128	
			559.272218	
Ani s 2.0101 (Q9NJA9)	HQDVCLDYTEQIEQLQK	1074.007318	1394.679801	39
			1279.652858	
			1116.589529	
	FEQQTIELSNK	668.840799	932.504737	24
			804.446159	
			703.398481	
	YQLAQQLEESR	682.843873	960.474499	24
			889.437386	
			761.378808	
	IDELLVELEAAQR	749.909213	1028.573485	27
			915.489421	
			816.421007	
	QSEQIIQLQANLEDTQR	1007.513625	1315.660068	36

			1187.601491	
			1074.517427	
	DLEVALDEETR [a]	645.314614	833.399937	23
			762.362824	
			649.27876	
	ADLSVQLIALTDR [a]	707.898648	1028.609871	25
			929.541457	
			801.482879	
	ADQAESSLNLIR [a]	658.843873	931.520721	23
			802.478128	
			715.4461	
	ISDLTSINSNLTAIK [a]	795.440877	1060.5997	28
			973.567671	
			860.483607	
	LLQDDFESER [a]	626.296224	897.358467	22
			782.331523	
			667.30458	
	QAEADLEEAHVR [a]	684.33113	968.479585	24
			853.452642	
			740.368578	
	LTAALADAEAR [a]	551.29857	745.383893	19
			632.299829	
			561.262716	
	LQDAECATDSQIESNR [a]	918.905061	1120.522906	33
			1049.485792	
			948.438114	
Ani s 3.0101 (Q9NAS5)	AQEDLSTANSNLEEK	824.886663	1005.48473	29
			904.437051	
			833.399937	
	IVELEEELR [a]	565.308603	917.457452	20
			788.414859	
			675.330795	
	ANTVESQLK [a]	495.266738	804.446159	17
			703.398481	
			604.330067	
	SLEVSEEK [a]	460.732197	720.341026	16
			591.298432	
			492.230018	
	LEEATHTADESER [a]	744.334066	1045.454492	26
			944.406814	
			807.347902	

	VQEAEAEVAALNR [a]	700.36243	972.510885	25
			843.468292	
			772.431178	
	EDSYEEQJR [a]	584.759474	924.442137	21
			837.410108	
			674.34678	
	LEDELVHEK [a]	556.285128	869.436323	20
			754.40938	
			625.366787	
	SISEELDQTFQELSGY [a]	923.423078	1187.521509	33
			1072.494566	
			944.435989	
Ani s 4.0101 (Q14QT4)	YTLEILVK	489.797143	815.523681	17
			714.476003	
			601.391939	
	DGDHQDLCTVTIWQK	908.42015	1263.640185	33
			1148.613242	
			1035.529178	
	WENFEEVK	540.753464	894.420339	19
			765.377745	
			651.334818	
Ani s 5.0101 (A1IKL2)	DLDAWVDTLGGDYK	784.367378	1153.552415	28
			967.473102	
			868.404688	
	AHEEAVAK	427.721967	783.399543	15
			646.340632	
			517.298038	
	IAEDDSLNGIQK	651.830431	874.462872	23
			759.435929	
			672.403901	
	GIGPAVPQ	369.710871	568.308937	13
			511.287474	
			414.23471	
	AFFELLK	434.2524	796.4604	15
			649.3919	
			502.3235	
Ani s 6.0101 (A1IKL3)	CDNGEPVICTYQCEHR	1019.414216	1462.65658	37
			1365.603816	
			1266.535402	
			1153.451338	
	YGTEFCSSLLQSCSASTGAILPLR	1309.633088	1460.752589	47

Ani s 7.0101 (A9XBJ8)			1332.694011	
			385.25578	
	NTNYASLPQVLK	674.366984	855.529829	24
			784.492716	
			697.460687	
			584.376623	
	GLLETCTEK	525.760432	767.323996	18
			638.281402	
			537.233724	
	YGAEFCQR	515.724183	810.356299	18
			739.319185	
			610.276592	
	YGQEFCNK	523.224016	825.355964	18
			697.297387	
			568.254794	
	LAATCSTETNTPLPQQDPWR	1143.544599	1452.723003	41
			1351.675324	
			1237.632397	
			1136.584719	
			926.447891	
			458.251029	
	YGDDFCLSLGK	637.78972	939.460429	23
			824.433486	
			677.365072	
	YGIEFCNR	529.739834	838.387599	19
			725.303535	
			596.260942	
	LGQGTCQQAVK	595.303329	891.435277	21
			834.413814	
			733.366135	
	SQVAMATCQK	562.2654	609.3644	20
			738.3273	
			607.2868	
Ani s 8.0101 (A7M6Q6)	FLDGADQATK	533.264196	690.341694	19
			633.32023	
			562.283117	
	DAFAALAQTFK	591.811313	778.445765	21
			707.408652	
			594.324588	
	QVEDAVQQFVNDHPAIK	969.489421	1296.669511	35
			1168.610933	

			1040.552356<br>428.286745	
	EVLAAQQAAEEEHK	776.883726	1069.490878<br>941.4323<br>813.373723	28
	LVAALPPDAQK	561.829506	839.462144<br>768.42503<br>655.340966<br>558.288202	20
	ADAELTAIADDASLTLAAK	930.48347	1188.647044<br>1075.56298<br>1004.525866	33
	IVQTFESLPPAVK	714.908484	1088.598637<br>987.550959<br>840.482545<br>511.323859<br>414.271095	25
Ani s 9.0101 (B2XCP1)	QLANGAPDK [b]	457.240524	672.33113<br>601.294016<br>487.251088<br>359.192511	16
	TEAEIEAQIEQWVASK [b]	916.457255	1159.610599<br>1088.573485<br>960.514908	33
	GGAVQAEFNK [b]	510.75908	736.36243<br>608.303852<br>537.266738	18
	AEAAHQASLTR [b]	577.799268	883.47444<br>812.437326<br>675.378414	20
	LSAIASNR [a, b]	416.237784	631.352199<br>560.315085<br>447.231021	14
	QLAAAFQALDPAVK [b]	721.903733	1059.583322<br>988.546208<br>841.477794<br>414.271095	26
Ani s 10.0101 (D2K835)	ANEQAAEQQNIGVGGPGPVK	982.495235	1250.685161<br>1122.626583<br>994.568006<br>497.308209	35

			343.233982	
Ani s 11.0101 (E9RFF3)	FPVGGPGPIISGDGVNVWQK [c]	1012.533632	1412.753241	36
			1315.700477	
			1202.616413	
			1089.532349	
	QVNIPPPFIR [c]	590.84549	839.513785	21
			726.429721	
			629.376957	
			532.324194	
	FPVGGPGPIISGDGVNVLQK	976.036007	1493.832219	35
			1339.757992	
			1242.705228	
			1129.621164	
			1016.5371	
Ani s 11.0201 (E9RFF5)	FPVGGPGPIISGDGVNVWQK [c]	1012.533632	1412.753241	36
			1315.700477	
			1202.616413	
			1089.532349	
	QVNIPPPPFIR [c]	639.371872	936.566549	23
			823.482485	
			726.429721	
			629.376957	
			532.324194	
	GPLPIGGPGPVVSGSGIGR	837.470303	1406.775039	30
			1082.595283	
			985.542519	
			928.521056	
Ani s 12.0101 (E9RFF6)	EQCIESQIVIR [b]	687.855926	957.572757	24
			844.488693	
			715.4461	
	YGENCAELIK [b]	598.784438	976.476808	21
			847.434215	
			733.391287	
	QCVTITGAPPVTIGGSGQYR [b]	1031.522938	1231.642962	37
			1134.590198	
			1037.537434	
	DECAPSEAAEK [b]	603.750792	802.394124	21
			731.35701	
			634.304246	
	DENSLEVFPETFSQCFK [b]	1038.964587	1290.582336	37
			1143.513922	

			1046.461158	
Ani s 13.0101 (A0A221C790)	EGYTAADVQK [b]	541.261653	732.388644	19
			631.340966	
			560.303852	
	QGQNILLACHVVCATYDDR [b]	1117.028062	1466.615109	40
			1395.577996	
			1235.547347	
	QAWLEIGK [b]	472.763634	816.461415	16
			745.424302	
			559.344989	
	EFSSEITK [b]	470.73474	664.351196	16
			577.319168	
			490.287139	
	QNGVDLYK [b]	468.742899	694.377017	16
			637.355553	
			538.287139	
	ENYTAEDVQK [a, b]	598.775124	790.394124	21
			689.346445	
			618.309331	
	DDIHLPQAQWHEFWK [b]	975.468292	1356.648381	35
			1259.595618	
			1131.53704	
			1060.499926	
	LFAEYLDQK [b]	563.792589	866.425424	20
			795.38831	
			666.345717	
	SHSHLTEDEK [b]	591.772916	871.415587	21
			734.356676	
			621.272612	
	HSWTTIGEEFGHEADK [b]	922.415922	1118.474893	33
			1061.45343	
			932.410836	
	AGHHEGEHK [b]	501.231021	873.396189	17
			736.337278	
			599.278366	
Ani s 14.0101 (A0A0S3Q267)	LPPGIVGCAQTTDPVVQCR	1034.519341	1274.615761	37
			1203.578647	
			1075.52007	
			758.39777	
	YSESFCNR	531.719098	812.335563	19
			683.29297	

			596.260942	
	VTGTDIPAFSR	582.306395	906.467957	20
			805.420279	
			690.393336	
			577.309272	
	CSQQCDGWR	598.732214	949.394475	21
			821.335898	
			693.27732	
	QSGLVALK	408.252903	600.407923	14
			543.38646	
			430.302396	
	TNPLNAQCR	537.261464	858.425047	19
			761.372283	
			648.288219	
	CTTAIPYLK	533.78371	805.481817	19
			704.434138	
			633.397024	
			520.31296	

Supplementary Figure 1S. Standard curves of monoclonal antibody UA3-based ELISA for the detection of Ani s 7 allergen in biological samples. The graphs show the optical densities obtained testing two-fold dilutions *A. simplex* s.l. crude worme protein extract (CrP) ranging from 2.5 to 160 µg/mL (A), and truncated recombinant Ani s 7 (tAni s 7) ranging from 0.02 to 10 µg/mL (B). The horizontal dashed lines represent the estimated cut-off of the assay (2.5 µg/mL for *A. simplex* CrP, and 40 ng/mL for tAni s 7).

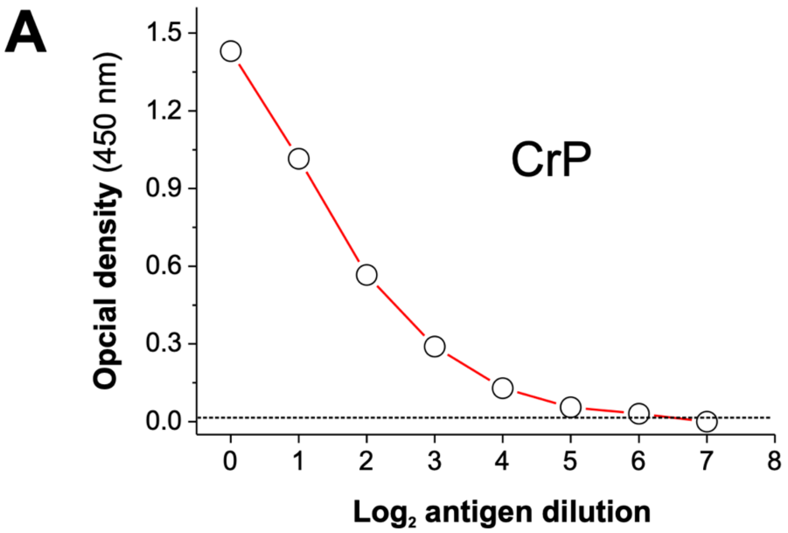

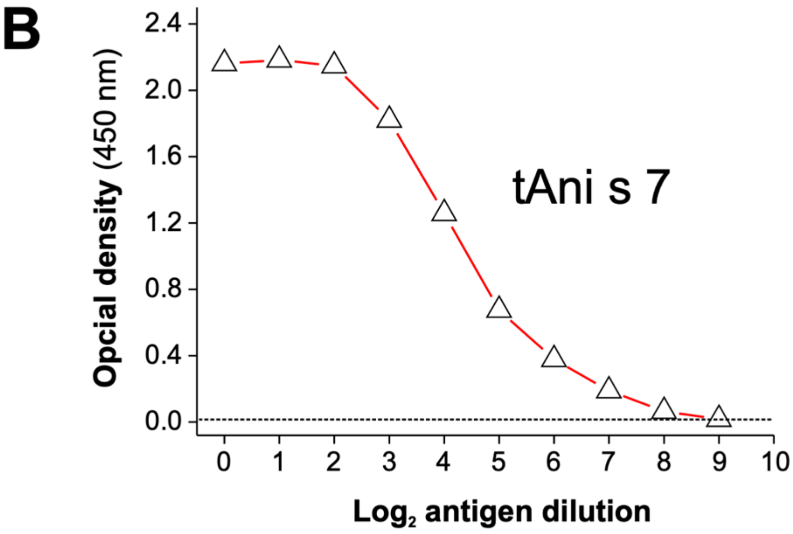

Supplementary Figure S2. St A) Full-length SDS-PAGE gel showing the protein profiles present in whole extracts from the *A. simplex* s.s. and *P. decipiens* s.s. after blotting on PVDF membranes (B). The remaining proteins in the gel were stained with BlueSafe (Nzytech, Lisboa, Portugal). After blotting, the PVDF membranes were blocked, cut into strips, and stored at -20 ºC before revealing of Ani s 7 and Ani s 7-like antigens with mAb UA3 in the absence (-) or presence (+) of the synthetic peptide P3 which inhibits the epitope recognized by mAb UA3. The rectangles in the figure show the cropped regions used to make Figure 3.

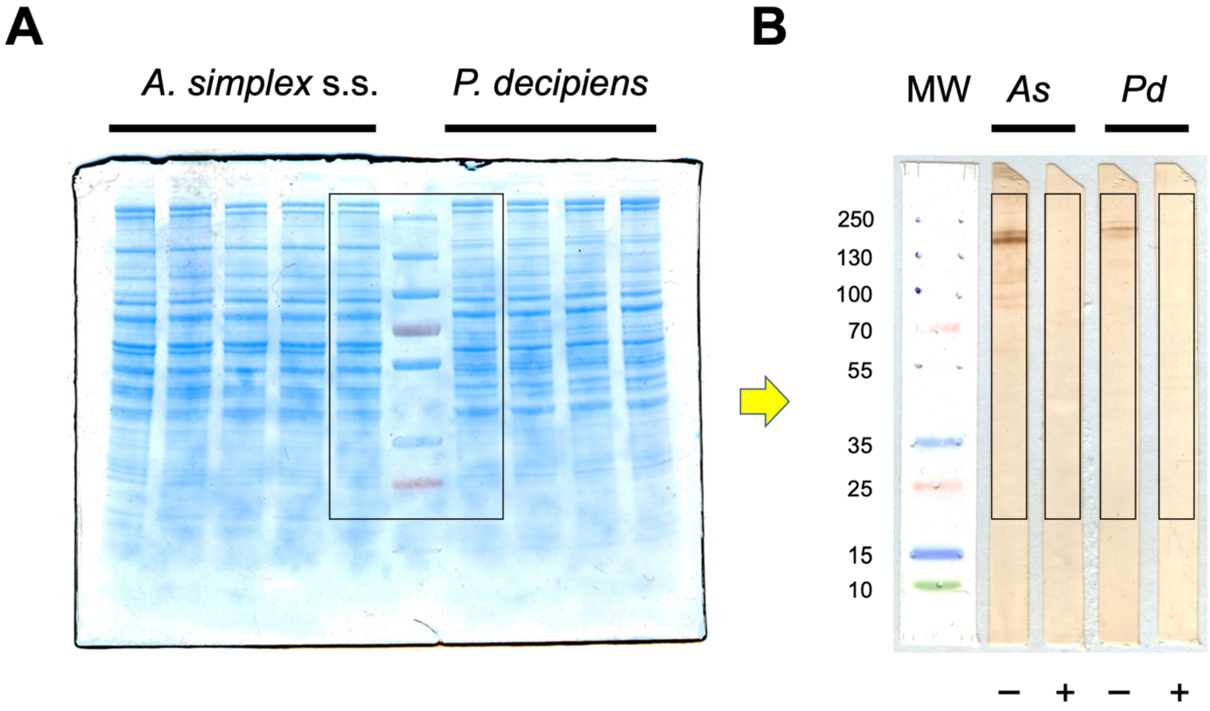

Supplement: Supplementary file 1 — Supplementary Information. [file 41598_2022_8113_MOESM1_ESM.pdf]
